# Supplementary material for: EEG-Based Prediction of Stress Responses to Naturalistic Decision-Making Stimuli in Police Cadets
Source: Sensors (Basel). 2025 Sep 22;25(18):5925. doi: 10.3390/s25185925 (PMC12473167; doi:10.3390/s25185925)
Supplement: Supplementary file 1 [file sensors-25-05925-s001.zip › sensors-3855321-supplementary.pdf]

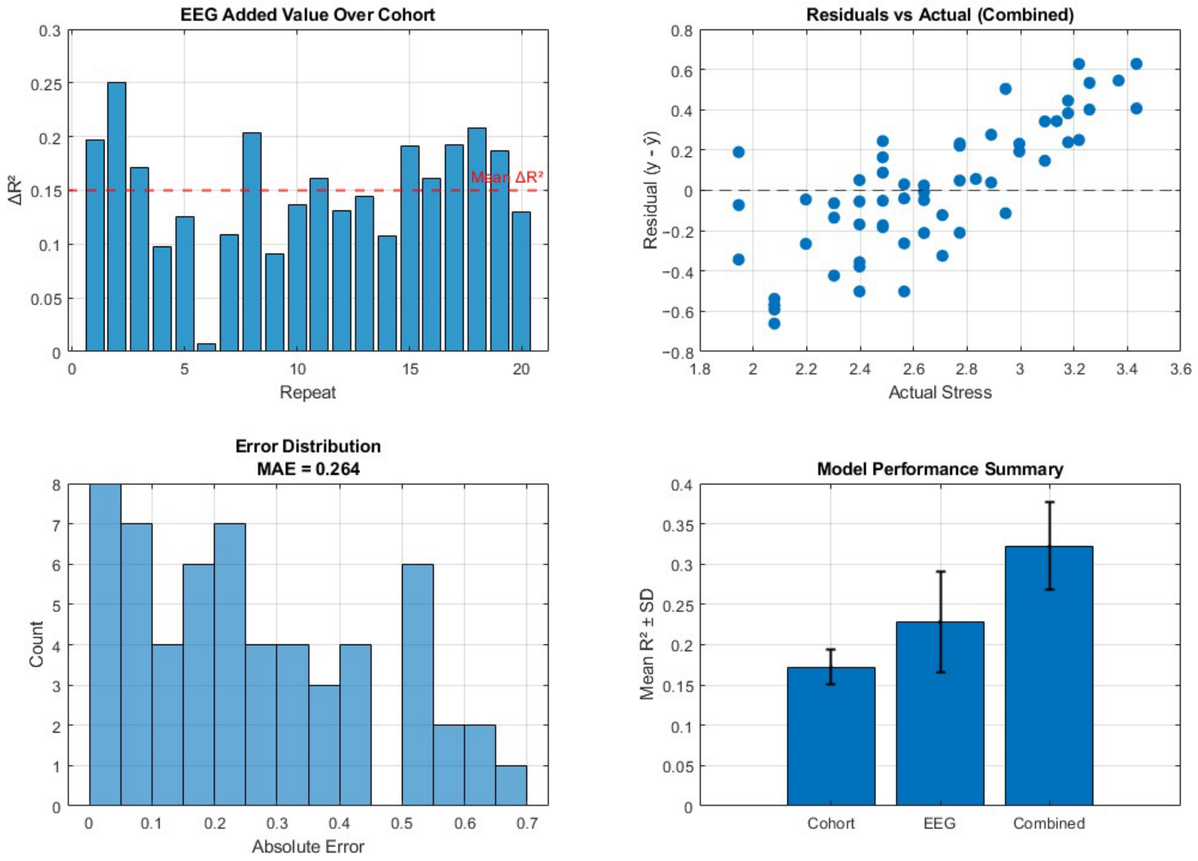

Figure S1: Cross-validated stress prediction statistics.

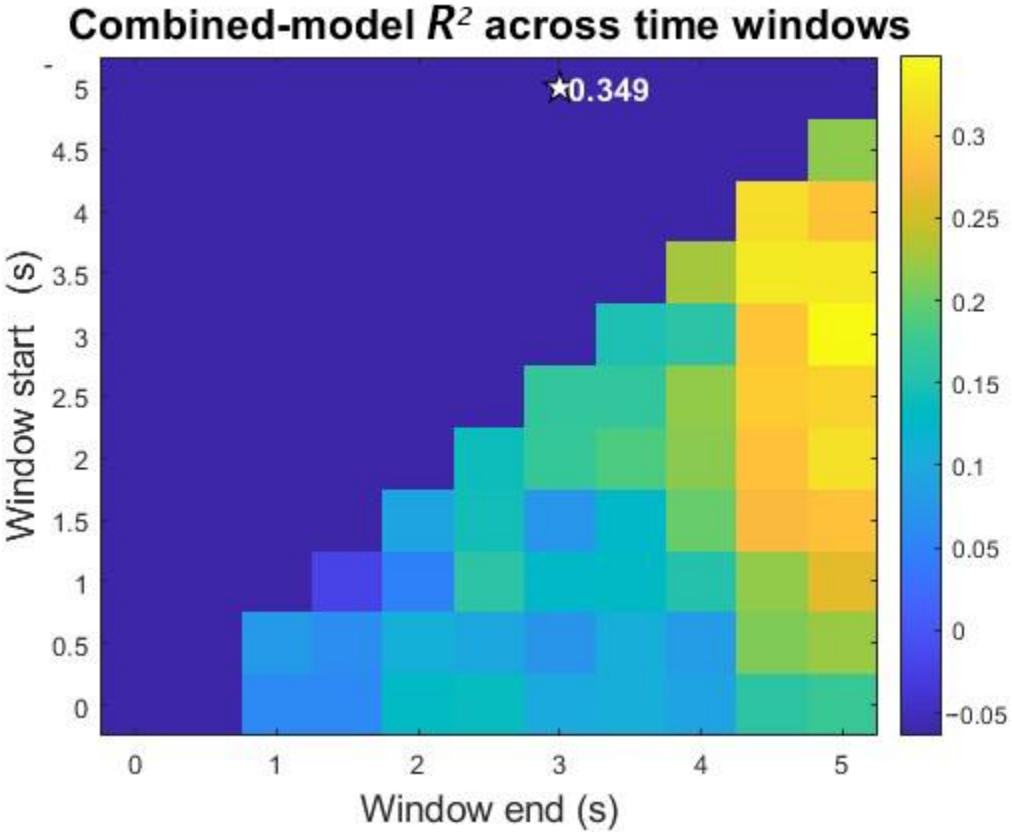

Figure S2: Optimal time window hyperparameter search.
